# Current Targeted Therapy for Metastatic Colorectal Cancer

**DOI:** 10.3390/ijms24021702

**Published:** 2023-01-15

**Authors:** Tomokazu Ohishi, Mika K. Kaneko, Yukihiro Yoshida, Atsuo Takashima, Yukinari Kato, Manabu Kawada

**Affiliations:** 1 Institute of Microbial Chemistry (BIKAKEN), Numazu, Microbial Chemistry Research Foundation, 18-24 Miyamoto, Numazu-shi 410-0301, Shizuoka, Japan; 2 Institute of Microbial Chemistry (BIKAKEN), Laboratory of Oncology, Microbial Chemistry Research Foundation, 3-14-23 Kamiosaki, Shinagawa-ku 141-0021, Tokyo, Japan; 3 Department of Antibody Drug Development, Tohoku University Graduate School of Medicine, 2-1 Seiryo-machi, Aoba-ku, Sendai 980-8575, Miyagi, Japan; 4 Department of Thoracic Surgery, National Cancer Center Hospital, 5-1-1 Tsukiji, Chuo-ku 104-0045, Tokyo, Japan; 5 Department of Gastrointestinal Medical Oncology, National Cancer Center Hospital, Tsukiji, Chuo-ku 104-0045, Tokyo, Japan; 6 Department of Molecular Pharmacology, Tohoku University Graduate School of Medicine, 2-1 Seiryo-machi, Aoba-ku, Sendai 980-8575, Miyagi, Japan

**Keywords:** colorectal cancer, targeted therapy, clinical trial

## Abstract

Colorectal cancer (CRC) is the third most common type of cancer and the second leading cause of cancer deaths worldwide. Surgery or surgery plus radiotherapy and/or chemotherapy for patients with metastatic CRC (mCRC) were accepted as the main therapeutic strategies until the early 2000s, when targeted drugs, like cetuximab and bevacizumab, were developed. The use of targeted drugs in clinical practice has significantly increased patients’ overall survival. To date, the emergence of several types of targeted drugs has opened new possibilities and revealed new prospects for mCRC treatment. Therapeutic strategies are continually being updated to select the most suitable targeted drugs based on the results of clinical trials that are currently underway. This review discusses the up-to date molecular evidence of targeted therapy for mCRC and summarizes the Food and Drug Administration-approved targeted drugs including the results of clinical trials. We also explain their mechanisms of action and how these affect the choice of a suitable targeted therapy.

## 1. Introduction

Colorectal cancer (CRC) arises from the epithelial cells lining the colon or rectum of the gastrointestinal tract and is the third most common cancer type among men and women in the United States [1]. CRC is also the third most commonly diagnosed cancer and was the second leading cause of cancer deaths in 2020 worldwide [2]. Surgery or surgery plus radiotherapy and chemotherapy in the adjuvant setting has improved the survival of patients with CRC, and the 5-year survival rate for CRC is 65%. However, because this falls to 15% for metastatic CRC (mCRC), the development of new therapeutic approaches to mCRC are critical [1,3].

Although complete surgical resection of the tumor and its metastatic sites improves overall survival (OS) in patients with CRC, approximately 25% of CRCs are diagnosed at an advanced stage with metastases in distant organs, which is difficult to manage surgically [4]. Unresectable advanced or recurrent CRC is treated with chemotherapy along with targeted therapy and/or radiotherapy to reduce the tumor size and prolong patient survival [5]. Regarding chemotherapy and targeted therapy, there are several first-line therapeutic options, and understanding the gene mutation status in CRC and resistance mechanisms is crucial to choose the best therapeutic option [6]. Notably, on rare occasions, the treatment may facilitate tumor downstaging, thereby improving the opportunity for resection.

Cytotoxic chemotherapy remains the standard treatment strategy for mCRC. Fluoropyrimidines play an important role as the backbone of combination regimens. Chemotherapy, such as FOLFOX (fluorouracil, leucovorin, and oxaliplatin), FOLFIRI (fluorouracil, leucovorin, and irinotecan), or FOLFOXIRI (fluorouracil, leucovorin, oxaliplatin, and irinotecan), combined with or without targeted drugs (anti-epidermal growth factor receptor [EGFR] antibody or anti-vascular endothelial growth factor [VEGF] antibody) is considered the first-line treatment for mCRC [7]. When chemotherapy is used, the doctor should give the patient as much information as possible about side effects because their severity depends on various factors, such as the type of cytotoxic drugs used and the duration of treatment [8,9]. Therefore, the active management of side effects is crucial so that the patient can continue chemotherapeutic treatment.

Several targeted drugs have been developed and studied. These drugs target the molecules involved in tumorigenesis and their related signaling pathways in cancer cells that make them different from normal cells [10]. Additionally, the tumor microenvironment, including blood vessels in the tissue surrounding the tumor and immune cells, are also affected by these targeted drugs to impede tumor growth and improve antitumor immune surveillance and attack [11]. The major types of targeted drugs are monoclonal antibodies and small molecule inhibitors. Such drugs are advantageous because, unlike chemotherapy, they can be chosen based on the molecular characteristics of tumor types [12]. However, a large number of patients experience disease progression even after receiving standard regimens. In the future, personalized medicine, in which drugs and drug combinations are optimized based on each patient’s available data including their genetic and epigenetic features and alterations, will improve the efficacy of treatments, reduce side effects, and benefit cancer patients [13,14].

This review summarizes the up-to-date evidence of clinical successes using targeted therapies to treat patients with mCRC. The molecular mechanisms of action and how these affect the choice of a suitable targeted therapy are also discussed.

## 2. mCRC Treatment Strategies

In the 1990s, fluorouracil-based chemotherapy improved the OS of patients with mCRC to 14 months. Later, the additional combination of leucovorin and oxaliplatin (FOLFOX) prolonged the OS to 19.5 months [15,16]. In 2004, the first Food and Drug Administration (FDA)-approved targeted drug was the anti-EGFR antibody cetuximab [17]. Since then, many targeted drugs for mCRC have been approved by FDA (Table 1).

**Figure 1:**
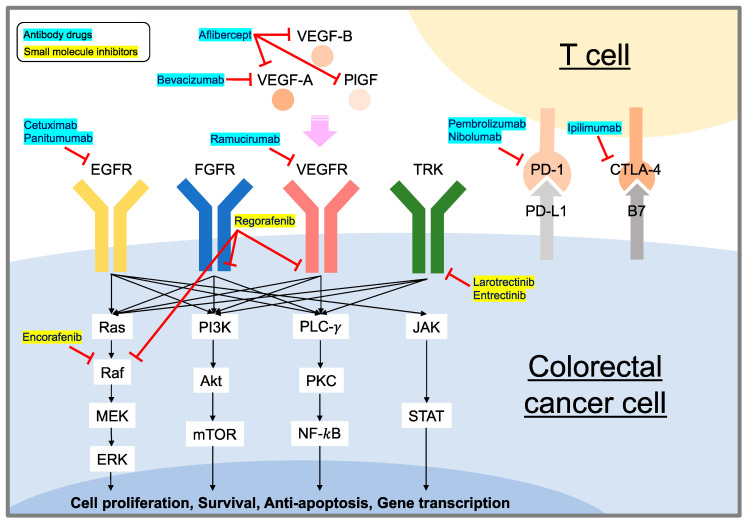
Comprehensive overview of FDA-approved targeted drugs for mCRC. Abbreviations: EGFR: epidermal growth factor receptor; FGFR: fibroblast growth factor receptor; VEGFR: vascular endothelia growth factor receptor; TRK: tropomyosin receptor kinase; VEGF: vascular endothelial growth factor; PlGF: placenta growth factor; PD-1: programmed cell death 1; PD-L1: programmed cell death ligand 1; CTLA-4: cytotoxic T-lymphocyte-associated protein-4; B7: CD80/CD86; MEK: mitogen-activated protein kinase; ERK: extracellular signal-regulated kinase; PI3K: phosphoinositide 3-kinase; mTOR: mechanistic target of rapamycin; PLC-γ: phospholipase C-γ; PKC: protein kinase C; NF-*k*B: nuclear factor-kappa B; JAK: Janus kinase; STAT: transducer and activator of transcription.

The progression and spread of mCRC involves mediation with receptors in several signaling pathways. These include EGFRs, fibroblast growth factor receptors (FGFRs), vascular endothelia growth factor receptors (VEGFRs), and tropomyosin receptor kinases (TRKs) [29,30]. Furthermore, tumor cells express B7-1 (CD80)/B7-2 (CD80) and programmed cell death ligand 1 (PD-L1), which bind to cytotoxic T-lymphocyte-associated protein-4 (CTLA-4) and programmed cell death 1 (PD-1), respectively, on T cells to escape immune surveillance [30]. Therefore, these molecules and their related pathways must be inhibited by targeted drugs (Figure 1).

## 3. EGFR-Targeting Strategy

### 3.1. Molecular Mechanism of EGFR Signaling

EGFR is a member of the ErbB family of receptors, a subfamily of four receptor tyrosine kinases, including EGFR (ErbB-1), HER2 (ErbB-2), HER3 (ErbB-3), and HER4 (ErbB-4). It consists of extracellular, transmembrane, and intracellular domains and regulates cell proliferation, survival, differentiation, and migration [31]. The binding of ligands to the extracellular domain of EGFR promotes receptor dimerization, activating downstream signaling pathways, such as RAS/Raf/mitogen-activated protein kinase (MEK)/extracellular signal-regulated kinase (ERK), phosphoinositide 3-kinase (PI3K)/Akt, Janus kinase (JAK)/signal transducer and activator of transcription (STAT), and phospholipase C (PLC)-γ/protein kinase C (PKC), leading to the activation of gene transcription and playing key roles in cancer initiation and progression [32,33,34,35]. Several tumors, including mCRCs, overexpress EGFR, and the aberrant EGFR signaling is associated with poor prognosis. Thus, this receptor is a promising target for mCRC treatment [36].

### 3.2. Cetuximab and Panitumumab

Cetuximab and panitumumab are FDA-approved agents targeting EGFR (Table 1 and Figure 1). They are both distinct monoclonal antibodies and are used in monotherapy or in combination therapy with chemotherapy to treat patients with RAS wild-type mCRC [37]. Multiple reports have demonstrated that responses to either cetuximab or panitumumab occur exclusively in patients with mCRC without mutations in KRAS and NRAS codons 12 and 13 of exon 2, codons 59 and 61 of exon 3, and codons 117 and 146 of exon 4 [38].

Cetuximab is a chimeric mouse-human monoclonal antibody of the IgG_1_ subclass. It had the highest capacity to stimulate antibody-dependent cell-mediated cytotoxicity (ADCC) compared with other isotypes (such as IgG_2_, IgG_3_, and IgG_4_) [39,40]. It is thought that ADCC is mainly mediated by natural killer cells or macrophages, and it is one of the important modes of action of therapeutic antibodies [41]. Cetuximab showed great potential in an initial phase II clinical trial [42]. This was also confirmed by a randomized phase II trial (BOND trial) of cetuximab plus irinotecan or single irinotecan, which reported an OS of 22.9 months (218 subjects) vs. 10.8 months (111 subjects), respectively, in irinotecan-refractory patients [43] (Table 2).

Panitumumab is a fully human monoclonal IgG_2_ antibody. Whereas cetuximab, a chimeric mouse-human monoclonal antibody, might induce immunogenic reactions, there is less fear of this happening with panitumumab [46]. In fact, panitumumab was reported to show a lower risk of hypersensitivity reactions than cetuximab [47]. On the other hand, unlike cetuximab, panitumumab does not induce ADCC [48]. In a randomized phase III trial (PRIME trial), panitumumab plus FOLFOX4 showed improved progression-free survival (PFS) and OS of patients with mCRC compared with FOLFOX4 alone [44] (Table 2).

Importantly, cetuximab and panitumumab recognize and bind to domain III of EGFR and are effective in patients with wild-type RAS mCRC; both agents showed similar OS in a phase III trial (ASPECCT trial) [49,50]. Panitumumab shows effectiveness following cetuximab failure, and cetuximab is effective following panitumumab failure, indicating that the mechanisms of action of these two agents differ [51,52].

Although cetuximab and panitumumab have been used to treat mCRC, their use is limited to patients with wild-type RAS because patients with RAS mutations do not benefit from anti-EGFR treatment [53,54,55,56,57,58,59,60,61,62,63,64,65]. Notably, although approximately 50% of patients with CRC harbor RAS mutations (KRAS: 36% and NRAS: 3%) [66], not all patients with KRAS mutations are resistant to EGFR-targeted therapy [67]. There are conflicting reports with respect to the KRAS codon G13D mutation. Several retrospective studies demonstrated that cetuximab confers clinical benefits to patients with KRAS codon G13D-mutated mCRC compared with patients harboring other KRAS mutations [68,69]. A cell line from a tumor with a KRAS codon G12V mutation was unresponsive to cetuximab and panitumumab, whereas cell lines from a tumor with a KRAS codon G13D mutation showed an intermediate responsive to cetuximab and panitumumab in comparison with resistant KRAS codon mutation G12V and wild-type cells [70]. De Roock et al. investigated the association of response and survival between patients with KRAS codon G13D and other mutations (data set of 579 patients with chemotherapy-refractory CRC treated with cetuximab). They found that cetuximab treatment was associated with longer PFS and OS [71]. Although this finding was obtained from a retrospective study, in a randomized phase II study, Nakamura et al. showed that cetuximab-based treatment benefited patients with chemotherapy-resistant, refractory KRAS codon G13D-mutated mCRC [72]. Because of the limitations of the low number of patients with KRAS mutations in the datasets, further clinical studies with larger sample sizes are necessary to evaluate the differences in the efficacy of the EGFR-targeting strategy for the patients with the KRAS codon G13D mutation and those with KRAS mutations other than codon G13D.

It is important to note that tumor-sidedness has been identified as an important prognostic factor found in several clinical studies [73,74]. It has been reported that patients with wild-type KRAS/RAS having right-sided tumors are associated with worse prognosis to anti-EGFR treatment than left-sided tumors regardless of the first-line treatment given [74,75]. In addition, retrospective analyses have revealed that wild-type RAS with right-sided mCRC also leads to poor prognosis in second and later line anti-EGFR treatments than left-sided tumors [76]. Since the left and right colon have different embryologic origins, the left colon is from the foregut and right from midgut, this may explain the difference in treatment effects. Based on the results and randomized clinical trial results above, patients with right-sided mCRC with wild-type KRAS/NRAS/BRAF mutations should use anti-EGFR containing chemotherapy regimens [77,78].

Rechallenge with anti-EGFR treatment in patients who have responded to first-line regimens containing an EGFR targeting drug is emerging as a valid therapeutic strategy in patients with mCRC. In a phase II single-arm clinical trial (CRICKET trial), Cremolini et al. demonstrated that cetuximab rechallenge strategy is beneficial in patients with RAS and BRAF wild-type mCRC with acquired resistance to first-line cetuximab-based treatment (median PFS, 4.0 vs. 1.9 months, *p* < 0.001) [79]. In this trial, liquid biopsies are used to assess the circulating tumor DNA (ctDNA), and it is found that only patients with no RAS and BRAF mutations detected in their ctDNA may have a clinical benefit by the rechallenge with anti-EGFR treatment. In a single arm, open-label phase II study (CHRONOS trial), compared with standard third-line treatments, liquid biopsy-driven anti-EGFR rechallenge with panitumumab maximizes the therapeutic effects and avoids adverse effects [80].

## 4. VEGF/VEGFR Targeting Strategy

Angiogenesis is the process whereby new vessels are formed or reformed from existing vessels, and tumor angiogenesis plays an important role in tumor growth. The VEGF/VEGFR signaling pathway is recognized as one of the most predominant factors contributing to tumor angiogenesis, which participates in the multiple processes of tumor progression by activating host vascular endothelial cells [81]. 

Although, a VEGF/VEGFR-targeted strategy has been used in the patients with CRC with or without RAS mutations, patients with left-sided mCRC and right-sided mCRC with KRAS/NRAS/BRAF mutations should be considered for a VEGF/VEGFR-targeted drug containing chemotherapy regimens.

### 4.1. Molecular Mechanism of VEGF/VEGFR Signaling

VEGF family proteins and VEGFRs are key factors of tumor growth and metastasis that regulate normal and pathological tumor angiogenesis, leading to the activation of several signaling pathways [81]. The VEGF family consists of five members (VEGF-A, B, C, D, and placenta growth factor [PlGF]) that bind to endothelial cells through VEGFRs, including VEGFR-1, VEGFR-2, and VEGFR-3 [82]. While VEGF-A, VEGF-B, and PlGF mainly induce angiogenesis, VEGF-C and VEGF-D tend to regulate lymphangiogenesis [83]. VEGF-A, VEGF-B, and PlGF bind to VEGFR-1; VEGF-A, VEGF-C, and VEGF-D bind to VEGFR-2; and VEGF-C and VEGF-D bind to VEGFR-3; respectively, leading to various biological responses [84]. VEGFs cause VEGFR dimerization, which activates intrinsic tyrosine kinase, leading to the activation of signaling pathways, such as RAS/Raf/MEK/ERK, PI3K/Akt, and PLC-γ/PKC, to enhance tumor angiogenesis and proliferation. Among them, VEGFR-1 and VEGFR-2, which are common receptors for VEGF-A, are considered promising targets against cancer in clinical settings [85,86].

### 4.2. Bevacizumab

Bevacizumab is a humanized anti-VEGF-A monoclonal IgG_1_ antibody that inhibits VEGF-A binding to VEGFR-1 and VEGFR-2, and it is approved by the FDA to treat mCRC [87] (Table 1 and Figure 1). Hurwitz et al. demonstrated that relative to placebo plus IFL (irinotecan, fluorouracil, and leucovorin), the addition of bevacizumab to IFL when treating patients with mCRC significantly improved the 1-year survival rate (74.3% in 402 subjects vs. 63.4% in 411 subjects), OS (20.3 vs. 15.6 months), PFS (10.6 vs. 6.2 months), and response rate (RR) (44.8% vs. 34.8%) [88] (Table 3). In a randomized phase III trial (AVEX study), the addition of bevacizumab to capecitabine showed a tolerable safety profile and efficient administration and significantly improved OS (20.7 months in 140 subjects vs. 16.8 months in 140 subjects) and PFS (9.1 vs. 5.1 months) relative to single capecitabine in elderly patients (aged > 70 years) [89] (Table 3). Furthermore, in a randomized phase III trial (TRIBE trial), Cremolini et al. showed that a combination regimen of bevacizumab with the FOLFOXIRI showed better efficacy than that with FOLFIRI (OS: 31.0 vs. 25.8 months, *p* = 0.125; PFS: 12.1 vs. 9.7 months, *p* = 0.006; RR: 65% vs. 53%, *p* = 0.006; respectively) [90].

Although some clinical trials using bevacizumab with chemotherapy showed partial improvement in OS or PFS, bevacizumab in combination with chemotherapy is similarly effective in patients with KRAS wild-type and KRAS mutant mCRC [95]. In addition, Yoshimatsu et al. showed that there was no difference in therapeutic effect of bevacizumab for mCRC patient with RAS wild-type and RAS mutant [96]. Although both the EGFR and VEGF/VEGFR signaling pathways have been identified as possible therapeutic targets to treat patients with KRAS wild-type mCRC, data from several clinical trials (FIRE-3 trial and PEAK trial) shows that anti-EGFR treatment (cetuximab or panitumumab) appears superior to anti-VGFR treatment (bevacizumab) [77,78].

Importantly, Grothey et al. performed two large observational cohort studies and showed that after failure of bevacizumab combination chemotherapy for first-line treatment, continued bevacizumab beyond initial progression (BBP) improves OS and post progression survival [97,98]. In addition, during a randomized phase III trial (ML18147), relative to chemotherapy alone, second-line changing chemotherapy with bevacizumab at the same dose as used in the first-line treatment beyond disease progression improved median OS (11.2 months in 409 subjects vs. 9.8 months in 411 subjects) and PFS (6.9 vs. 4.67 months) [99]. From several other clinical studies including ARIES [100], RAISE [94], and VELOUR [101], BBP might have clinical benefits in patients who have experienced disease progression during a first-line bevacizumab containing chemotherapy.

### 4.3. Aflibercept

Aflibercept (also known as ziv-aflibercept) is a soluble molecule composed of the critical ligand-binding domains of human VEGFR-1 and VEGFR-2 fused with the Fc fragment of human IgG_1_. It functions as a decoy receptor by binding to VEGF-A, VEGF-B, and PlGF [102,103] (Table 1 and Figure 1). Aflibercept binds to VEGF-A with higher affinity and a faster association rate than bevacizumab [103]. In a randomized phase III trial (VELOUR trial), relative to placebo plus FOXFIRI, aflibercept plus FOXFIRI regimen improved OS (13.5 months in 612 subjects vs. 12.06 months in 614 subjects), PFS (6.9 vs. 4.67 months), and RR (19.8% vs. 11.1%) in patients with mCRC who progressed after receiving an oxaliplatin-based regimen [91] (Table 3). From these results, the FDA-approved aflibercept for the treatment of mCRC when given in combination with the FOLFIRI in 2012. However, in a randomized phase II trial (AFFIRM study), adding aflibercept to first-line modified FOLFOX6 (mFOLFOX6) did not show significant efficacy (aflibercept plus mFOLFOX6 in 119 subjects vs. mFOLFOX6 in 116 subjects, PFS: 8.48 vs. 8.77 months, RR: 49.1% vs. 45.9%). Use as a second-line treatment after progression following first-line treatment, aflibercept in combination with FOLFIRI showed efficacy following bevacizumab plus FOLFOXIRI for unresectable or mCRC in single arm phase II trials [104]. Therefore, the use of aflibercept-based regimens is recommended in second-line settings.

### 4.4. Ramucirumab

Ramucirumab is a fully human anti-VEGF-A monoclonal IgG_1_ antibody that inhibits VEGFR-2 and its downstream angiogenesis pathways. It was approved by the FDA for second-line use in combination with FOLFIRI for patients with mCRC who progressed during or after treatment with bevacizumab, oxaliplatin, and fluoropyrimidine [105]. In a second-line randomized phase III study (RAISE), relative to the placebo, ramucirumab in combination with FOLFIRI significantly improved OS (13.3 months in 536 subjects) vs. (11.7 months in 536 subjects) and PFS (5.7 vs. 4.5 months) with FOLFIRI [94] (Table 3). This was also confirmed by a retrospective study of ramucirumab plus FOLFIRI as a second- or later line therapy, which reported a positive impact on survival outcomes, with the PFS on second-line ramucirumab of 5.4 months (26 subjects) being equivalent to that observed in the RAISE trial [106]. Additionally, another retrospective study of ramucirumab plus FOLFIRI as second-line therapy reported an OS of 17.0 months (74 subjects) and PFS of 6.2 months (74 subjects), which was also equivalent to the RAISE trial [107].

### 4.5. Regorafenib

Regorafenib is a multikinase inhibitor that inhibits multiple intracellular and membrane-bound receptor tyrosine kinases, including VEGFR and FGFR involved in the regulation of tumor angiogenesis. It was approved by the FDA to treat previously treated patients with mCRC [108] (Table 1 and Figure 1). In a clinical phase III trial (CORRECT trial), Grothey et al. demonstrated that compared with the placebo, regorafenib significantly improved the OS (6.4 months in 505 subjects vs. 5.0 months in 255 subjects) and PFS (1.9 vs. 1.7 months) in treatment-refractory mCRC [92] (Table 3). This was also confirmed by a randomized phase III trial (CONCUR trial) of regorafenib plus best supportive care (BSC) or placebo plus BSC, which reported an OS of 8.8 months (138 subjects) vs. 6.3 months (68 subjects) and PFS of 3.2 vs. 1.7 months [93] (Table 3). Tyrosine kinase inhibitors (TKIs) have become one of the standard treatment regimens for patients with non-small-cell lung cancers [109]. Several TKIs have been tested for the patients with mCRC in clinical settings over recent years.

### 4.6. Fruquintinib

Fruquintinib is a potent and selective small molecule tyrosine kinase inhibitor of VEGFR 1, 2, and 3 [110]. It was approved in China for use in the treatment of mCRC. The FDA granted a fast track designation for the development of fruquintinib for the treatment of patients with mCRC who have received prior fluoropyrimidine-, oxaliplatin-, and irinotecan-based chemotherapy; EGFR-targeted therapy; and VEGF-targeted therapy. In a randomized, multicenter, phase III FRESCO trial conducted in China (NCT02314819), fruquintinib treatment in patients with mCRC who progressed after standard second-line therapy showed significant improvement in the PFS and OS [111]. Furthermore, in a randomized phase III FRESCO-2 trial (NCT04322539), with a total of 687 patients with refractory mCRC for more than third-line treatment, fruquintinib treatment showed significant PFS and OS [112]. These results indicate the clinical benefit of Fruquintinib for the patients with mCRC beyond third-line therapy.

## 5. Immune Checkpoint Targeting Immunotherapies

Immunotherapy is a novel treatment option against several types of tumors. Tumor immunotherapy induces an immune cell-mediated immune response through the neoantigen expressed on a broad range of tumors [113]. The success of tumor immunotherapy in achieving long-lasting antitumor responses has demonstrated that immune cells, mainly T cells, could be utilized to eliminate tumor cells [114].

Microsatellite instability (MSI) is an indicator of defective DNA mismatch repair (dMMR) and an MSI/dMMR status is observed in approximately 5% of mCRC cases [115]. Because MMR pathways are responsible for correcting DNA replication errors, MSI-high tumors carry various somatic mutations, leading to a high neoantigen exposure that favors the initiation of an antitumor immune response [116,117]. Therefore, MSI-high tumors respond well to immunotherapy.

### 5.1. Molecular Mechanism of Immune Checkpoints

Programmed cell death-1 (PD-1) and CTLA-4 are expressed on the surface of activated immune cells, including T cells, and are key immune checkpoint molecules that inactivate immune cells through distinct mechanisms [118]. PD-1 inhibits T cell response via interaction with its two ligands, PD-L1 and PD-L2, to mediate an inhibitory signal in T cells, which suppresses cellular and humoral immune responses [119]. Blockage of PD-1 or PD-L1 can inhibit the checkpoint and induce T cell activation to drive immunity [120,121].

CTLA-4 is expressed on activated T cells and regulatory T cells (Tregs). It inhibits T cell response by interacting with B7-1 and B7-2 ligands to provide an inhibitory signal in T cells [122]. Inhibition of CTLA-4 binding to these ligands results in the reactivation and proliferation of T cells and decreases immunosuppressive Tregs, leading to increased activation of the immune system in the tumor microenvironment [123].

### 5.2. Pembrolizumab, Nivolumab, and Ipilimumab

Pembrolizumab is a humanized IgG_4_ anti-PD-1 monoclonal antibody and nivolumab is a human IgG_4_ anti-PD-1 monoclonal antibody. They have been approved by the FDA for patients with multiple tumors, including MSI-high/dMMR mCRC [124] (Table 1 and Figure 1). In a phase II study (NCT01876511), pembrolizumab treatment in patients with MSI-high/dMMR mCRC in second-line settings showed an objective RR (ORR) of 52% (40 subjects), disease control rate of 82%, 2-year OS of 72%, and 2-year PFS of 59% [125] (Table 4). Furthermore, in an open-label phase III trial (KEYNOTE-177), pembrolizumab as first-line treatment for patients with MSI-high/dMMR mCRC was superior to chemotherapy with respect to PFS (16.5 months in 153 subjects vs. 8.2 months in 154 subjects) and ORR (43.8% vs. 33.1%). Importantly, treatment-related adverse effects of grade 3 or higher occurred in 22% of the patients in the pembrolizumab-treated group compared with 66% in the chemotherapy-treated group [126] (Table 4). In addition to pembrolizumab, in an open-label phase II trial (CheckMate 142), second-line treatment with nivolumab in patients with MSI-high/dMMR mCRC showed an ORR of 31% (74 subjects) and disease control for 12 weeks or longer of 69% [127] (Table 4). From these results, pembrolizumab and nivolumab are thought to be effective and show a manageable safety profile in patients with MSI-high/dMMR mCRC.

Ipilimumab is a fully human IgG_1_ anti-CTLA-4 monoclonal antibody. It has been approved by the FDA for combination therapy with nivolumab in patients with MSI-high/dMMR mCRC after progression following chemotherapy [25] (Table 1 and Figure 1). In an open-label phase II trial (CheckMate-142), relative to nivolumab, nivolumab in combination with ipilimumab in patients with MSI-high/dMMR mCRC improved 1-year OS (85% in 119 subjects vs. 73% in 74 subjects), ORR (55% vs. 31%), and disease control for 12 weeks or longer (80% vs. 69%) [128] (Table 4). These results indicated that the combination of nivolumab and ipilimumab showed a favorable impact on the quality of life for patients with MSI-high/dMMR mCRC.

## 6. NTRK Signaling Pathway and Targeting Strategy

The neurotrophic tropomyosin receptor kinases (NTRK) family consists of three members (NTRK1, NTRK2, and NTRK3) that code for TRKA, TRKB, and TRKC, respectively, and they are mainly expressed in neural and neuronal tissues [129]. They perform biological functions by homodimerization, which activates their downstream pathways, including RAS/Raf/MEK/ERK, PI3K/Akt, and PLC-γ/PKC, leading to the activation of gene transcription, cell survival, and progression [130,131]. Multiple NTRK fusions are one of the most common oncogenic events, leading to constitutive activation of their downstream pathways [132]. Besides NTRK, anaplastic lymphoma kinase (ALK) and c-ros oncogene 1 (ROS1) fusions also occur in CRC. ALK and ROS1 also encode tyrosine kinases that are constitutively activated by fusion, leading to the activation downstream signaling pathways for tumor cell growth and progression [133,134]. Their gene rearrangements are thought to be mutually exclusive in lung cancers despite ALK and ROS1 tyrosine kinase domains sharing high homology [135]. Although these fusions occur in less than 2.5% of CRC cases, they are considered oncogenic drivers and a potential target for mCRC treatment [136,137].

### Larotrectinib and Entrectinib

Larotrectinib and entrectinib are first-generation TRK inhibitors. They were approved by the FDA for patients with solid tumors that harbor NTRK gene fusions [138] (Table 1). Larotrectinib is a selective inhibitor of TRK that was tested in three phase I/II clinical trials of 153 cancer patients carrying NTRK fusions (age: 48–67 years) and showed an impressive ORR of 79% and good tolerability [139] (Figure 1).

Entrectinib is a potent TRK, ROS1, and ALK kinase inhibitor that was tested in three phase I/II clinical trials of 54 cancer patients carrying NTRK fusions (age: 1 month–84 years) and showed an impressive ORR of 57% and good tolerability [137] (Figure 1).

## 7. BRAF Signaling Pathway and Targeting Strategy

BRAF is a downstream effector of RAS in the RAS/Raf/MEK/ERK signaling pathway and is a well-known oncogenic driver [140]. Mutations in BRAF V600E are present in approximately 10% of mCRCs and associated with chemotherapy resistance and worse prognosis [141,142]. Interestingly, non-V600 BRAF mutation was independently and significantly associated with improved OS [142]. Given that monotherapy with a BRAF-targeting tyrosine kinase inhibitor for patients with mCRC has failed, some combination regimens have been tested in multiple clinical trials [143].

### Encorafenib

Encorafenib is a kinase inhibitor that targets BRAF V600E as well as wild-type BRAF and shows more prolonged pharmacodynamic activity than other BRAF inhibitors [144] (Table 1 and Figure 1). In an open-label phase III trial (BEACON), compared with the control (cetuximab plus chemotherapy), encorafenib in combination with cetuximab showed a significantly improved median OS (8.4 months in 220 subjects vs. 5.4 months in 221 subjects) and confirmed RR (20% vs. 2%) [45] (Table 2). Additionally, in BEACON trial, compared with the control, a triple regimen containing encorafenib, cetuximab, and a MEK inhibitor, binimetinib also showed a significantly improved median OS (9.0 months in 224 subjects vs. 5.4 months in 221 subjects) and confirmed RR (24% vs. 2%) [45] (Table 2). From these results, in 2020, the FDA-approved encorafenib for the treatment of mCRC with a BRAF V600E mutation after prior therapy.

## 8. Concluding Remarks and Future Perspectives

This review focused on FDA-approved targeted drugs that are currently available to treat patients with mCRC. Our objective was to address the efficacy of these treatments. We included most clinical trials that were associated with FDA approvals, reviewed trial-associated publications, and explored other relevant clinical trials. 

Recent advancements in sequencing technologies have led to a better understanding of comprehensive genomic and proteomic alterations in mCRC, which helps to choose the correct treatment strategy. Although the latest therapeutic strategies have achieved substantial progress in patients with mCRC, emerging resistance and lack of predictive biomarkers to current targeted therapies remains a major problem in clinical settings. Further understanding of resistance mechanisms and exploration of biomarkers that can predict the treatment sensitivity and efficacy/toxicity are required to test new evidence-based therapeutic strategies, expand treatment options, and realize personalized medicine [145,146,147,148]. 

To further improve the efficacy of treatments, innovations, such as CRC multicellular 3D models, patient-derived xenograft models, and single-cell sequencing strategy, and applying new antibody-based therapies strategies, including chimeric antigen receptor T cell therapy [149], antibody drug conjugates [150], radioimmunotherapy [151], and photoimmunotherapy [152], may provide a survival benefit for patients with mCRC. To date, we have developed several cancer-specific monoclonal antibodies, including anti-EGFR antibodies using the CasMab method [153,154,155,156,157,158,159,160,161,162,163]. Currently, we are trying to further develop these antibodies for the above-mentioned antibody-based therapy.

Several promising targets including HER2, KRAS codon G12C mutation, and RET have been revealed and their targeted therapies are under development and/or clinical evaluation [164,165,166]. Continued comprehensive understanding of mCRC pathology and molecular drivers, and developing novel therapeutic approaches are necessary to further refine the treatment of mCRC. While validated targets, including RAS, BRAF, TRK, and MSI/dMMR, are important to choose the correct therapeutic strategy for patients with mCRC [167]. This is because disclosure of additional targets may lead to the discovery of potential predictive biomarkers of treatment response and the correct selection of patients. Further studies, including clinical trials, hold promise for improved outcomes and progress toward personalized medicine for mCRC.

## Figures and Tables

**Table 1:** FDA-approved targeted drugs for mCRC.

Year Approved by the FDA	Drugs	Targets	Drug Details	Ref
2004	Cetuximab	EGFR	Chimeric mouse/human mAb (IgG_1_)	[18]
	Bevacizumab	VEGF-A	Humanized mAb (IgG_1_)	[18]
2006	Panitumumab	EGFR	Fully human mAb (IgG_1_)	[19]
2012	Aflibercept	VEGF-A, VEGF-B, PlGF	Fusion protein which consists of the binding portions of VEGF from VEGF-1 and 2 fused to the Fc portion ofimmunoglobulin G_1_ (IgG_1_)	[20]
	Regorafenib	VEGFR, FGFR, KIT, PDGFR, BRAF	Small molecule inhibitor of membrane-bound and intracellular receptor tyrosine kinases	[21]
2015	Ramucirumab	VEGFR-2	Fully human mAb (IgG_1_)	[22]
2017	Pembrolizumab	PD-1	Humanized mAb (IgG_4_)	[23]
	Nivolumab	PD-1	Fully human mAb (IgG_4_)	[24]
2018	Ipilimumab	CTLA-4	Fully human mAb (IgG_1_)	[25]
	Larotrectinib	TRK	Small molecule of tyrosine kinase inhibitor	[26]
2019	Entrectinib	TRK, ALK, ROS1	Small molecule of tyrosine kinase inhibitor	[27]
2020	Encorafenib	BRAF (V600E-mutant)	Small molecule kinase inhibitor	[28]

Abbreviations: EGFR: epidermal growth factor receptor; VEGF: vascular endothelial growth factor; PlGF: placenta growth factor; VEGFR: vascular endothelia growth factor receptor; FGFR: fibroblast growth factor receptor; KIT: mast/stem cell growth factor receptor; PDGFR: platelet-derived growth factor receptor; BRAF: v-Raf murine sarcoma viral oncogene homolog B1; PD-1: programmed cell death 1; CTLA-4: cytotoxic T-lymphocyte-associated protein-4; TRK: tropomyosin receptor kinase; ALK: anaplastic lymphoma kinase; ROS1: c-ros oncogene 1; mAb: monoclonal antibody.

**Table 2:** Selected clinical trials for EGFR (± BRAF)-targeting drugs.

Targeted Drugs	Study	Phase	Study Regimen	Number	Results	Ref
Cetuximab	BOND	II	Cetuximab + Irinotecan /Irinotecan (Refractory to Irinotecan) (Third-line)	218/111	OS: 22.9/10.8 months (*p* = 0.007) Disease control: 55.5%/32.4% (*p* < 0.001)	Cunningham et al. [43]
Panitumumab	PRIME	III	Panitumumab + FOLFOX4 /FOLFOX4 (First-line)	325/331	OS: 23.8/19.4 months (*p* = 0.03) PFS: 10.0/8.6 months (*p* = 0.01)	Douillard et al. [44]
Cetuximab + Encorafenib	BEACON	III	Cetuximab + Encorafenib /Cetuximab + Chemotherapy ^1^ (Second- or later-line)	220/221	Median OS: 8.4/5.4 months (*p* < 0.001) Confirmed RR: 20%/2% (*p* < 0.001)	Kopetz et al. [45]
Cetuximab + Encorafenib + Binimetinib	BEACON	III	Cetuximab + Encorafenib + Binimetinib /Cetuximab + Chemotherapy ^1^ (Second- or later-line)	224/221	Median OS: 9.0/5.4 months (*p* < 0.001) Confirmed RR: 26%/2% (*p* < 0.001)	Kopetz et al. [45]

^1^ The investigators’ choice of either cetuximab and irinotecan or cetuximab and FOLFIRI (folinic acid, fluorouracil, and irinotecan. Abbreviations: OS: overall survival; PFS: progression-free survival; RR: response rate.

**Table 3:** Selected clinical trials for VEGF/VEGFR-targeting drugs.

Targeted Drugs	Study	Phase	Study Regimen	Number	Results	Ref
Bevacizumab	Clinical study	III	Bevacizumab + IFL/Placebo + IFL (First-line)	402/411	OS: 20.3/15.6 months (*p* < 0.001) PFS: 10.6/6.2 months (*p* < 0.001) RR: 44.8%/34.8% (*p* = 0.004) 1-year survival rate: 74.3%/63.4% (*p* < 0.001)	Hurwitz et al. [88]
Bevacizumab	AVEX	III	Bevacizumab + Capecitabine/Capecitabine (First-line)	140/140	OS: 20.7/16.8 months (*p* = 0.18) PFS: 9.1/5.1 months (*p* < 0.001)	Cunningham et al. [89]
Aflibercept	VELOUR (NCT00561470)	III	Aflibercept + FOLFILI/Placebo + FOLFILI (Second-line)	612/614	OS: 13.5/12.06 months (*p* = 0.0032) PFS: 6.9/4.67 months (*p* < 0.0001) RR: 19.8%/11.1% (*p* = 0.0001)	Van Cutsem et al. [91]
Regorafenib	CORRECT (NCT01103323)	III	Regorafenib/Placebo (Third- or later-line)	505/255	OS: 6.4/5.0 months (*p* = 0.0052) PFS: 1.9/1.7 months (*p* < 0.0001)	Grothey et al. [92]
Regorafenib	CONCUR (NCT01103323)	III	Regorafenib/Placebo (Third- or later-line)	138/68	OS: 8.8/6.3 months (*p* = 0.00016) PFS: 3.2/1.7 months (*p* < 0.0001)	Li et al. [93]
Ramucirumab	RAISE (NCT01183780)	III	Ramucirumab + FOLFIRI/Placebo + FOLFIRI (Second-line)	536/536	OS: 13.3/11.7 months (*p* = 0.0219) PFS: 5.7/4.5 months (*p* < 0.0005)	Tabernero et al. [94]

Abbreviations: OS: overall survival; PFS: progression-free survival; RR: response rate.

**Table 4:** Selected clinical trials for immune checkpoint-targeting drugs.

Targeted Drugs	Study	Phase	Study Regimen	Number	Results	Ref
Pembrolizumab	Clinical study (NCT01876511)	II	Pembrolizumab (Second-line) (Patients with MSI-high/dMMR mCRC)	40	ORR: 52% Disease control rate: 82% 2-year OS: 72% 2-year PFS: 59%	Le et al. [125]
Pembrolizumab	KEYNOTE-177 (NCT02563002)	II	Pembrolizumab/Chemotherapy (First-line) (Patients with MSI-high/dMMR mCRC)	153/154	PFS: 16.5/8.2 months ORR: 43.8%/33.1%	Andre et al. [126]
Nivolumab	CheckMate-142 (NCT02060188)	II	Nivolumab (Second-line) (Patients with MSI-high/dMMR mCRC)	74	ORR: 31% Disease control for 12 weeks or longer: 69%	Overman et al. [127]
Nivolumab +Ipilimumab	CheckMate-142 (NCT02060188)	II	Nivolumab + Ipilimumab/Nivolumab (Second-line) (Patients with MSI-high/dMMR mCRC)	119/74	1-year OS: 85%/73% ORR: 55%/31% Disease control for 12 weeks or longer: 80%/69%	Overman et al. [128]

Abbreviations: ORR: objective response rate; OS: overall survival; PFS: progression-free survival.

## Data Availability

Not applicable.
